# Transient sex-related changes in the mice hypothalamo–pituitary–adrenal (HPA) axis during the acute phase of the inflammatory process

**DOI:** 10.1155/S0962935193000183

**Published:** 1993

**Authors:** T. Daneva, E. Spinedi, R. Hadid, M.-C. Jacquier, M. Giacomini, R. C. Gaillard

**Affiliations:** 1 Neuroendocrine Unit Division of Endocrinology Department of Medicine University Hospital Geneva 4 CH-1211 Switzerland; 2 Division of Endocrinology and Metabolism University Hospital (CHUV) Lausanne CH-1011 Switzerland

## Abstract

The potential role of endogenous sex hormones in regulating hypothalamo–pituitary–adrenal (HPA) axis function was investigated after a single injection of endotoxin in adult (8 week old) BALB/c mice of both sexes. The effect of LPS on plasma ACTH, corticosterone (B), testosterone and oestradiol (E) levels and on anterior pituitary (AP) ACTH and adrenal B contents at different times after treatment was studied. The results indicate that: (a) basal B but not ACTH plasma levels were significantly higher in female than in male mice; (b) LPS significantly increased both ACTH and B plasma levels over the baseline 2 h after injection, both hormone levels being higher in female than in male mice; (c) although plasma ACTH concentrations recovered the basal value at 72 h after LPS in animals of both sexes, plasma B levels returned to the baseline only at 120 h after treatment; (d) E plasma levels significantly increased 2 h after LPS and returned to the baseline at 72 h post-treatment, in both sexes; (e) at 2 h after LPS, testosterone plasma levels significantly decreased in male mice and increased in female mice, recovering the baseline level at 120 and 72 h after LPS, respectively; (f) AP ACTH content was similar in both sexes in basal condition and it was significantly diminished 72 h post-treatment without sex difference; whereas AP ACTH returned to basal content 120 h after LPS in males, it remained significantly decreased in females; (g) basal adrenal B content was higher in female than in male mice, and it significantly increased in both sexes 2 h post-LPS, maintaining this sex difference. Whereas adrenal B returned to basal content 72 h after treatment in male mice, it remained significantly enhanced up to 120 h post-LPS in female animals. The data demonstrate the existence of a clear sexual dimorphism in basal condition and during the acute phase response as well as in the recovery of the HPA axis function shortly after infection.


Mediators of Inflammation, 2, 123–127 (1993)

### Transient sex-related changes in the mice hypothalamo–pituitary–adrenal (HPA) axis during the acute phase of the inflammatory process

T. Daneva, E. Spinedi, R. Hadid, M.-C. Jacquier, M. Giacomini and R. C. Gaillard.*,CA

Neuroendocrine Unit, Division of Endocrinology, Department of Medicine, University Hospital, CH-1211 Geneva 4, Switzerland
*Present Address: Division of Endocrinology and Metabolism, University Hospital (CHUV), CH-1011 Lausanne, Switzerland

**THE potential role of endogenous sex hormones in regulating hypothalamo–pituitary–adrenal (HPA) axis function was investigated after a single injection of endotoxin in adult (8 week old) BALB/c mice of both sexes. The effect of LPS on plasma ACTH, corticosterone (B), testosterone and oestradiol (E) levels and on anterior pituitary (AP) ACTH and adrenal B contents at different times after treatment was studied. The results indicate that: (a) basal B but not ACTH plasma levels were significantly higher in female than in male mice; (b) LPS significantly increased both ACTH and B plasma levels over the baseline 2 h after injection, both hormone levels being higher in female than in male mice; (c) although plasma ACTH concentrations recovered the basal value at 72 h after LPS in animals of both sexes, plasma B levels returned to the baseline only at 120 h after treatment; (d) E plasma levels significantly increased 2 h after LPS and returned to the baseline at 72 h post-treatment, in both sexes; (e) at 2 h after LPS, testosterone plasma levels significantly decreased in male mice and increased in female mice, recovering the baseline level at 120 and 72 h after LPS, respectively; (f) AP ACTH content was similar in both sexes in basal condition and it was significantly diminished 72 h post-treatment without sex difference; whereas AP ACTH returned to basal content 120 h after LPS in males, it remained significantly decreased in females; (g) basal adrenal B content was higher in female than in male mice, and it significantly increased in both sexes 2 h post-LPS, maintaining this sex difference. Whereas adrenal B returned to basal content 72 h after treatment in male mice, it remained significantly enhanced up to 120 h post-LPS in female animals. The data demonstrate the existence of a clear sexual dimorphism in basal condition and during the acute phase response as well as in the recovery of the HPA axis function shortly after infection.**

**Key words:** ACTH, Endotoxic shock, Glucocorticoids, Neuroendocrine immunology, Sex steroids, Stress

### Introduction

Gram-negative bacterial sepsis is still one of the major causes of morbidity and mortality in humans and other animal species. The A portion of the bacerial lipopolysaccharide (LPS) interacts in the infected host[1] inducing the activation of immune-related cells, which in turn release cytokines.[2] The most important cytokines released during the acute phase of the endotoxic shock are interleukin-1 (IL-1) and tumour necrosis factor alpha (TNF);[3,4] these substances act synergistically, although through different mechanisms, to induce pathophysiological symptoms observed during infection.[3,5,6] It has been shown in different animal models that the infusion of IL-1 and TNF mimick the LPS induced effects during infection,[7–9] and that the treatment of mice with anti-TNF antibodies significantly diminishes the lethal effect of LPS in these animals.[10] Accumulated evidence indicates that both cytokines activate the hypothalamo–pituitary–adrenal (HPA) function by acting at multiple levels of this axis.[11–16]

There is also clinical and experimental evidence indicating that gonadal steroids can modulate the immunological function. It has been established previously that a sexual dimorphism exists in the immune response to different noxious substances.[17,18] Skin allograft rejection time in mice is longer in males than in females.[19] Male F1 N2B/N2W mice are less susceptible than females to the development of autoimmune lupus, but they will die if gonadectomized.[20] In addition, mitogen driven plaque forming cell response of B-lymphocytes *in vitro* is inhibited by androgens.[21] This evidence is strongly supported by the fact that

© 1993 Rapid Communications of Oxford Ltd specific receptors for sex hormones are present in organs responsible for the immune response.[22]

Taking into account the important role of the HPA axis after injury and the interactions between sex hormones and the immune function, the aim of the present study was to determine the time course and the sex dependence of the HPA axis response to a single injection of a sublethal dose of endotoxin in adult mice.

### Materials and Methods

*Animals:* Adult (8 week old) male and female BALB/c mice were obtained from Iffa-Credo (Paris, France) and housed for 2 weeks in plastic cages (6–8 mice per cage). They were maintained on a 12 h light-on period (07:00–19:00) with free access to purina chow and water. Animals were gently handled for 7 days prior to the day of the experiment.

*Experimental design:* In preliminary experiments it was determined that LPS (Sigma Chem. Co., St Louis, MO) injected i.p. at the dose of 2 mg/kg B.W. in adult mice of both sexes was sub-lethal and induced a maximal glucocorticoid release 2 h after treatment. Then, several groups (6–8 animals per group) of male and female (random cycling) mice were treated i.p. (at 09:00) with 50 $\mu l$ of vehicle alone (sterile saline solution, VEH) or containing the above mentioned dose of LPS. Different groups of LPS injected mice of both sexes were decapitated 2, 72 and 120 h after treatment and trunk blood was collected. Each three VEH treated mice of both sexes were also decapitated at the same times after injection and trunk blood was collected and, for convenience, they represent the sample time zero. Plasma samples were frozen ($-20°C$) until the measurement of ACTH (Nichols Institute Diagnostics, San Juan Capistrano, CA), corticosterone (B),[23] testosterone[24] and oestradiol (E) (Bio-Merieux, France) concentrations by specific immunoassays.

Immediately after decapitation the anterior pituitary (AP) and adrenal glands were dissected as reported previously.[23] Tissues were then transferred into plastic tubes containing 200 $\mu l$ of 0.1 M acetic acid and sonicated for 45 s, then centrifuged (at 10 000 × $g$ for 5 min) and the supernatants were kept frozen ($-20°C$) until further determination of AP ACTH and adrenal B contents by specific assays.

*Analysis of data:* Data were analysed by analysis of variance, followed by Fisher's test for comparison of different mean values.[25]

### Results

*Sex difference in the LPS stimulated ACTH and glucocorticoid release in plasma:* Figure 1 shows the time course of plasma ACTH levels during the endotoxic shock. Basal (sample time zero) plasma ACTH concentrations were similar in both sexes. Plasma ACTH concentrations were significantly ($p < 0.05$) increased over the baseline 2 h after LPS treatment in both groups of mice, although values reached in female mice were significantly ($p < 0.05$) higher than those attained in male animals. As is also shown in Fig. 1, plasma ACTH levels returned to baseline 72 h after LPS injection in both groups of mice.

Figure 2 shows plasma glucocorticoid levels before and at different times after endotoxin administration. Basal plasma B values were significantly ($p < 0.05$) higher in female than in male mice. Coinciding with the rise in plasma ACTH levels as a consequence of endotoxin administration, plasma B concentrations were significantly ($p < 0.05$) increased over the baseline 2 h after LPS in a dimorphic fashion, since values in female mice were significantly higher than in male animals. Figure 2 also shows that plasma B levels were still higher than baseline 72 h post-LPS and returned to basal levels 120 h after endotoxin treatment in animals of both sexes.

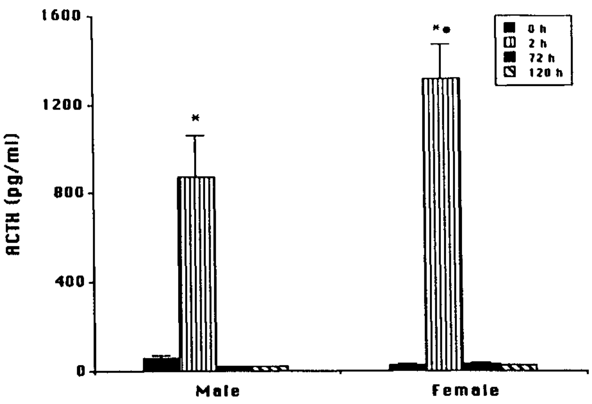

FIG. 1. Time-related effect of endotoxin (i.p., 2 mg/kg B.W.) on plasma ACTH levels in mice of both sexes. Bars represent the mean ± S.E.M. ($n = 6–8$ mice per group). *, $p < 0.01$ *vs.* the respective sample time zero. •, $p < 0.05$ *vs.* female values at 2 h after LPS.

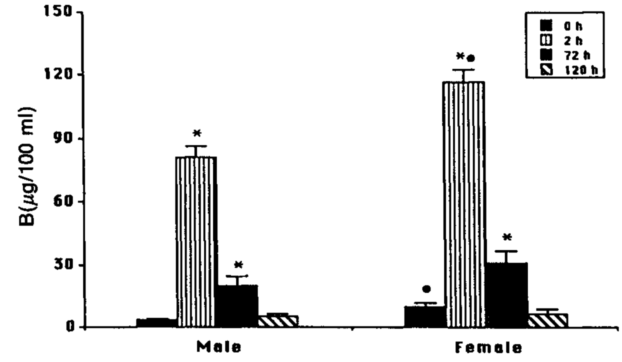

FIG. 2. Basal and LPS-induced corticosterone (B) release in plasma. Bars represent the mean ± S.E.M. ($n = 6–8$ mice per group). *, $p < 0.05$ or less *vs.* the respective sample time zero. •, $p < 0.05$ *vs.* male values at the same time interval.

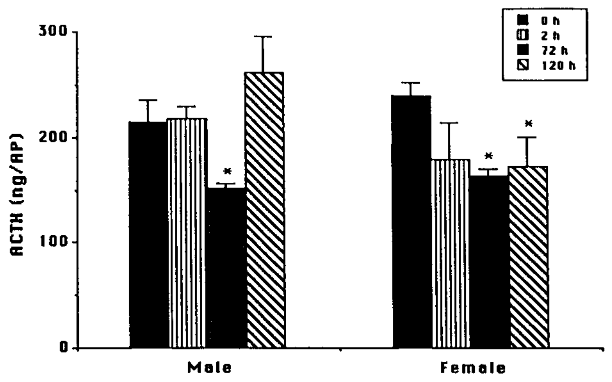

FIG. 3. Anterior pituitary gland (AP) ACTH content before and several times after endotoxin treatment in adult mice of both sexes. Bars represent the mean ± S.E.M. ($n = 6$–8 mice per group). *, $p < 0.05$ *vs.* the respective sample time zero.

*Effects of LPS and endogenous sex hormone environment on anterior pituitary ACTH and adrenal B contents:* Anterior pituitary ACTH content in both basal conditions and at different times after LPS, in male and female mice, are shown in Fig. 3. As shown, basal AP ACTH was similar in both groups of mice. The same figure shows that AP ACTH was significantly ($p < 0.05$) decreased at 72 h after endotoxin in animals of both sexes. Whereas male mice recovered basal AP ACTH content 120 h after treatment, anterior pituitary hormone content at this time was still significantly ($p < 0.05$) lower than the baseline in female animals.

The effect of LPS treatment on adrenal gland B content in male and female mice is shown in Fig. 4. Adrenal B content in basal conditions was significantly ($p < 0.05$) higher in female than in male animals. In both sexes, the adrenal glucocorticoid content was significantly ($p < 0.05$) increased over the baseline at 2 h after LPS; however, adrenal B content was significantly ($p < 0.05$) higher in female than in male mice. Figure 4 also shows that adrenal B content returned to basal values at 72 h after endotoxin treatment in male mice, whereas it remained significantly ($p < 0.05$) elevated over the baseline up to 120 h after endotoxin administration in female animals.

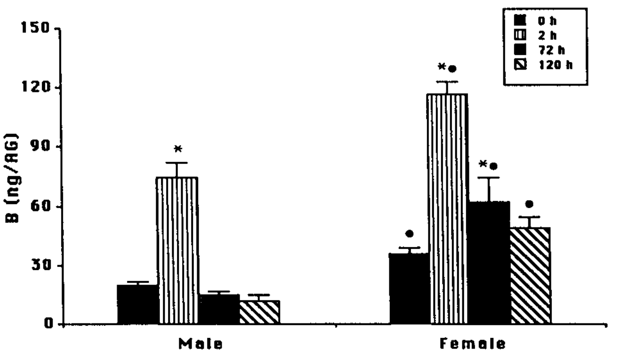

FIG. 4. Adrenal gland (AG) glucocorticoid (B) content before and after LPS treatment in male and female mice. Bars represent the mean ± S.E.M. ($n = 6$–8 mice per group). *, $p < 0.05$ or less *vs.* the respective sample time zero. •, $p < 0.05$ *vs.* male values at the same time interval.

**Table 1.** Plasma testosterone (To) and estradiol (E) levels before and several times after the administration (i.p.) of endotoxin (2 mg/kg B.W.) in adult mice of both sexes

| Steroid time | Male | | Female | |
|---|---|---|---|---|
| | To | E | To | E |
| 0 h | 4.6 ± 1.9 | 38.2 ± 4.5 | 0.34 ± 0.03 | 35.4 ± 3.5 |
| 2 h | 1.4 ± 0.3* | 60.4 ± 5.8* | 0.75 ± 0.11* | 90.4 ± 5.3*† |
| 72 h | 0.4 ± 0.1* | 19.4 ± 3.0 | 0.34 ± 0.02 | 33.7 ± 2.7† |
| 120 h | 5.7 ± 2.8 | 26.6 ± 3.9 | 0.35 ± 0.04 | 48.2 ± 3.1† |

To and E values are expressed in ng and pg per ml of plasma, respectively (mean ± S.E.M.; $n = 6$–8 mice per group). *, $p < 0.05$ *vs.* time zero values. †, $p < 0.05$ *vs.* male E values at the same time-interval.

*Endotoxin induced changes in plasma sex steroid hormone levels in male and female mice:* Table 1 shows plasma testosterone and E levels before and at different times after LPS administration in mice of both sexes. In male animals, basal plasma testosterone levels significantly ($p < 0.05$) decreased 2 h after LPS with minimal values at 72 h after endotoxin; then the baseline recovered at 120 h post-LPS. Conversely, in female mice, plasma testosterone levels significantly ($p < 0.05$) increased over the baseline at 2 h after endotoxin and returned to basal levels at 72 h after treatment.

On the other hand, plasma E values significantly ($p < 0.05$) increased over the baseline 2 h after endotoxin treatment in mice of both sexes, then returned to basal levels at 72 h after treatment. As seen in Table 1, plasma E levels in basal condition were of a similar magnitude in animals of both sexes; however, 2 h post-LPS, plasma E levels were significantly ($p < 0.05$) higher in female than in male animals and the same occurred 72 and 120 h after endotoxin treatment.

### Discussion

The present study clearly demonstrates a sexual dimorphism in the HPA response during both the acute and the recovering phases of endotoxic shock. This observation is in agreement with previous data indicating the existence of a sexual dimorphism in the immune response to different stimuli.[17,18] Although it has recently been reported that LPS treatment enhances HPA axis function in a sexually dimorphic fashion,[26] to our knowledge this is the first observation indicating a sex hormone basis for the transient changes in the neuroendocrine immune response during acute inflammation.

Our results indicate that: (a) there exists a sexual dimorphism in the mice HPA axis status in basal condition, such as higher plasma levels and adrenal content of corticosterone in female than in male animals; (b) the LPS induced ACTH and B secretion in plasma is higher in female than in male mice; (c) whereas ACTH plasma levels returned to baseline 72 h post-LPS, plasma B values remained elevated at this time post-treatment, regardless of the sex of the animals; (d) a significant decrease in AP ACTH was found in animals of both sexes at 72 h after LPS and this parameter returned to baseline at 120 h after treatment in males but not in females; (e) adrenal B content significantly increased 2 h after LPS treatment in animals of both sexes and with maintained sex difference, and returned to baseline values at 72 h after endotoxin administration in male but not in female animals (which only recovered basal adrenal glucocorticoid content 120 h after LPS).

Besides the above mentioned sex-related differences in the HPA axis function of the mice, an acute increase in plasma ACTH and B levels 2 h after LPS was observed, which is in agreement with earlier reports[27] and clearly indicates a stage of transient stress. As well as other stressor induced ACTH release, a decrease in AP ACTH 72 h after endotoxin was found with values returning to baseline at 120 h or more after treatment.[23] They probably reflect a stage of transient increase in AP ACTH release and thus adrenal B output (2 h after LPS) as a result of secreted cytokines from LPS activated immune cells.[2] Cytokines then could enhance hypothalamic CRH secretion[16,28,29] and thus stimulate AP ACTH synthesis to recover basal values (120 h or more). This possibility is even more clear in female mice, which showed a higher amount of ACTH and B secreted in plasma after endotoxin treatment, with AP ACTH still decreased up to 120 h after stress. The delayed recovering of AP ACTH after LPS treatment in female mice fully agrees with data obtained in adrenalectomized rats of both sexes, which indicate that 2 and 14 days after surgery AP ACTH was significantly lower in female than in male animals (unpublished results). Thus, a sex dependent change in the HPA axis response to this particular stress is indicated.

With respect to the sex related HPA axis response during the endotoxic shock, it has recently been reported[26] that bilateral gonadectomy influences the LPS stimulated TNF release in plasma 2 h after treatment; thus, a sexual difference in the stimulatory effect of TNF, or any other released cytokine after injury, on the different levels of the mice HPA axis must not be discarded.

Regarding the effect of endotoxin on adrenal glucocorticoid content, it is important to point out that at a very short time interval after LPS administration (2 h), which is coincident with the highest plasma ACTH and B levels, we found an enhanced adrenal B content in animals of both sexes, showing higher absolute values in female than in male mice. These data coincide with preliminary results from our group which indicate that orchidectomy leads to enhanced adrenal B content after LPS treatment when compared with the endotoxin effect on intact male mice. It could be speculated that the rapid LPS induced ACTH[30] or other POMC related peptide release from pituitary and/or extrapituitary[31,32] origin could be responsible for increased adrenal glucocorticoid synthesis and release. Although it has been shown that LPS did not affect *in vitro* adrenal B output,[26] it is not possible to exclude an *in vivo* effect of LPS on adrenal steroid synthesis.

With respect to the transient changes in circulating sex steroid levels after endotoxin administration, decreased testosterone and increased E plasma levels were found in male mice 2 h post-LPS. These data fully agree with other previous reports[33,34] and further suggest an increased conversion of testicular testosterone to E (aromatization) as a consequence of endotoxin administration.[35] It has recently been reported[26] that testosterone plays an important role in inhibiting LPS stimulated HPA immune axis function. Thus, it could be speculated that in males, having high basal plasma testosterone concentration, the increased testicular aromatization of the androgen takes place as a body's defence mechanism in order to allow an enhanced immune response shortly after infection.

To the authors' knowledge there are no data in the literature regarding plasmatic androgen and oestrogen levels during septic shock in female animals; however, it has been reported that an increase in androgen production resulted after different types of stress.[36] We found that endotoxin administration rapidly (2 h) increased plasma testosterone and E levels in female mice and these parameters were restored to the respective baseline 72 h post-treatment. It is already known that in normal females ovarian androgen production is very low and the adrenal gland substantially contributes to the total androgen production by a peripheral conversion of androstenedione to testosterone. LPS indirectly stimulates adrenal steroidogenesis[34] and thus could induce an increase in plasma testosterone levels 2 h after treatment; then, testosterone from both peripheral and ovarian (thecal) origin reaches the ovarian granulosa cell compartment to be further converted to E by LPS stimulated aromatase activity.

In summary, this study demonstrates a sexual dimorphism in the HPA axis function in both basal and LPS stimulated conditions. Endotoxin treatment induces transient changes in the HPA axis which are of a similar fashion to those described after several types of stress, and are of a quite different magnitude when male and female parameters are compared. These results strongly suggest that endogenous sex steroid hormones play an important regulatory role in HPA axis function under stress. The exact mechanism of action whereby sex steroid hormones modulate the neuroendocrine immune response shortly after infection remains to be determined.

### References

1. Kasper DL, Finberg RW. The bacteria. In: Samsters M, Talmage DW, Frank MM, Austen K, Claman HN, eds. *Immunological Diseases.* Boston-Toronto: Little, Brown and Comp., 1988; **1:** 803–832.
2. Dinarello CA, Mier JW. Lymphokines. *New Engl J Med* 1987; **317:** 940–945.
3. Rivier C, Chizzonite R, Vale W. In the mouse, the activation of the hypothalamopituitary–adrenal axis by a lipopolysaccharide (endotoxin) is mediated through Interleukin-1. *Endocrinology* 1989; **125:** 2800–2805.
4. Zuckerman SH, Shellhaas J, Butler LD. Differential regulation of lipopolysaccharide–induced interleukin 1 and tumor necrosis factor synthesis: effects of endogenous and exogenous glucocorticoids and the role of the pituitary-adrenal axis. *Eur J Immunol* 1989; **19:** 301–305.
5. Girardin E, Grau G, Dayer J-M, Roux-Lombard P, Lambert PH. Tumor Necrosis factor and interleukin-1 in the serum of children with severe infectious purpura. *New Engl J Med* 1988; **319:** 397–400.
6. Feuerstein G, Hallenbeck JM, Vanatta B, Rabinovici R, Perera PY, Vogel SN. Effect of gram-negative endotoxin on levels of serum corticosterone, TNF, alpha, circulating blood cells, and the survival of rats. *Circulatory Shock* 1990; **30:** 265–278.
7. Okusawa S, Gelfand JA, Connoly R, Dinarello CA. Interleukin-1 induces a shock-like state in rabbits. *J Clin Invest* 1988; **81:** 1162–1172.
8. Tracey KJ, Lowry SF, Fahey TJ, *et al.* Cachectin/TNF induces lethal shock and stress-hormone response in the dog. *Surg Gynecol Obstetrics* 1987; **164:** 415–422.
9. Tracey KJ, Beutler B, Lowry S, *et al.* Shock and tissue injury induced by recombinant human cachectin. *Science* 1986; **234:** 470–474.
10. Tracey KJ, Fong Y, Hesse DG, *et al.* Anti-cachectin/TNF monoclonal antibodies prevent septic shock during lethal bacteremia. *Nature* 1987; **330:** 662–664.
11. Besedovsky H, Del Rey A, Sorkin E, Dinarello CA. Immunoregulatory feed-back between Interleukin-1 and glucocorticoid hormones. *Science* 1986; **233:** 652–654.
12. Rivier C. Role of endotoxin and Interleukin-1 in modulation of ACTH, LH and sex steroid secretion. In: Porter JC, Jezova D, eds. *Circulating regulatory factors and neuroendocrine function.* New York: Plenum Press, 1990; 295–301.
13. Sharp BM, Shannon GM, Peterson PK, Newton R, Cheo C, McAllen K. Tumor Necrosis Factor is a potent ACTH secretagogue: comparison to Interleukin-1 beta. *Endocrinology* 1989; **124:** 3131–3133.
14. Gaillard RC, Turnill D, Sappino P, Muller AF. Tumor Necrosis Factor-alpha inhibits the hormone response of the pituitary gland to hypothalamic releasing factors. *Endocrinology* 1990; **127:** 101–106.
15. Harbour DV, Galin FS, Hughes TK, Smith EM, Blalock JE. Role of the leucocyte-derived pro-opiomelanocortin peptides in endotoxic shock. *Circulatory Shock* 1991; **35:** 181–191.
16. Bateman A, Singh A, Kral T, Solomon S. The immune-hypothalamo–pituitary–adrenal axis. *Endocrinol Rev* 1989; **10:** 92–114.
17. Grossman CJ. Are there underlying immune-neuroendocrine interactions responsible for the immunological sexual dimorphism? *Prog Neuroendocr Immunol* 1990; **3:** 75–82.
18. Grossman CJ. Interactions between the gonadal steroids and the immune system. *Science* 1985; **227:** 257–260.
19. Graff RJ, Lappe MA, Snell GD. The influence of the gonads and adrenal glands on the immune response to skin grafts. *Transplant* 1969; **7:** 105–109.
20. Roubinian JR, Talal N, Siiteri PK, Sadakin JA. Sex hormones model of autoimmunity in NZB/NZW mice. *Arthr Rheum* 1979; **22:** 1162–1168.
21. Stroeger ZM, Chiorazzi N, Lahita RG. Regulation of the immune response by sex hormones. *In vivo* effect of estradiol and testosterone on pokeweed mitogen-induced human B-cell differenciation. *J Immunol* 1988; **141:** 91–97.
22. Grossman CJ. Regulation of the immune system by sex steroids. *Endocrinol Rev* 1984; **5:** 435–455.
23. Spinedi E, Giacomini M, Jacquier MC, Gaillard RC. Changes in the hypothalamo–corticotrope axis after bilateral adrenalectomy: evidence for a median eminence site of glucocorticoid action. *Neuroendocrinology* 1991; **53:** 160–170.
24. Sizonenko PC, Paunier L. Hormonal changes in puberty. Correlation of plasma dehydroepiandtosterone, testosterone, FSH and LH with stages of puberty and bone age in normal boys and girls and in patients with Addison disease or hypogonadism or with premature or late adrenarche. *J Clin Endocrinol Metab* 1975; **41:** 894–904.
25. Zar JH. *Biostatistical Analysis.* Englewood Cliffs: Prentice-Hall, 1974.
26. Spinedi E, Suescun MO, Hadid R, Daneva T, Gaillard R. Effect of gonadectomy and sex hormone replacement therapy on the endotoxin-stimulated hypothalamo–pituitary–adrenal axis: evidence for a neuro-endocrine-immunological sexual dimorphism. *Endocrinology* 1992; **131:** 2430–2436.
27. Kiwao N, Seiji S, Chanho O. Significance of increased secretion of glucocorticoids in mice and rats injected with bacterial andotoxin. *Brain Behavior Immunity* 1987; **1:** 159–172.
28. Berkenbosch F, De Rijk R, Del Rey A, Besedovsky H. Neuroendocrinology of interluekin-1. In: Porter JC, Jezova D, eds. *Circulating Regulatory Factors and Neuroendocrine Function.* New York: Plenum Press, 1990; 303–314.
29. McCann SM, Rettori V, Milenkovic L, Jurcovicova J, Gonzalez MC. Role of monokines in the control of the anterior pituitary hormone release. In: Porter JC, Jezova D, eds. *Circulating regulatory factors and Neuroendocrine function.* New York: Plenum Press, 1990; 315–328.
30. Barbanel G, Solie MD, Dornand J, Mecauche M, Ixart G, Siaud P, Assenmache I. Temporal profiles on plasma ACTH and cytokines after endotoxin challenges in rats. IX *Int Congress of Endocr,* Nice, France, 1992; P-01.31.049 (Abstract).
31. Baumann JB, Eberle AN, Christen E, Ruch W, Girard J. Steroidogenic activity of highly potent melanotropic peptides in the adrenal cortex of the rat. *Acta Endocrinol (Copenh)* 1986; **113:** 396–402.
32. Martin LW, Lipton JM. Acute phase response to endotoxin: rise in plasma alpha-MSH and effects of alpha-MSH injection. *Am J Physiol* 1990; **259:** R768–R772.
33. Christeff N, Benassayag C, Carli-Vielle C, Carli A, Nunez EA. Elevated oestrogen and reduced testosterone levels in the serum of male septic shock patients. *J Steroid Biochem* 1988; **29:** 435–440.
34. Christeff N, Auclair MC, Benassayag C, Carli A, Nunez EA. Endotoxin-induced changes in sex steroid hormone levels in male rats. *J Steroid Biochem* 1987; **26:** 67–71.
35. Christeff N, Auclair MC, Dehennin L, Thobie N, Benassayag C, Carli A, Nunez EA. Effects of the aromatase inhibitor 4-hydroxy-androstenedione on the endotoxin-induced changes in steroid hormones in male rats. *Life Sci* 1992; **50:** 1459–1468.
36. Carlberg KA, Alvin BL. Effect of blood sampling technique and exercise on plasma androgens in female rats. *Med Sci Sport Exercise* 1992; **24:** 610–614.

ACKNOWLEDGEMENTS. This work was supported by grants from the Swiss National Science Foundation (31-29876.090), and from the Roussel Institute, Roussel Santé R et D, Paris, France.

**Received 31 December 1992; accepted in revised form 25 January 1993**
